# Metabolic Changes in Zebrafish Larvae Infected with *Mycobacterium marinum*: A Widely Targeted Metabolomic Analysis

**DOI:** 10.3390/metabo15070449

**Published:** 2025-07-04

**Authors:** Chongyuan Sima, Qifan Zhang, Xiaoli Yu, Bo Yan, Shulin Zhang

**Affiliations:** 1 Center for Tuberculosis Research, Shanghai Public Health Clinical Center, Fudan University, Shanghai 201508, China; cysima2025@163.com (C.S.); zqf17852980787@163.com (Q.Z.); 2 School of Life Science and Technology, Wuhan Polytechnic University, Wuhan 430023, China; yxll268@126.com; 3 Department of Immunology and Microbiology, Shanghai Jiao Tong University School of Medicine, Shanghai 200025, China

**Keywords:** metabolomic, zebrafish, *Mycobacterium marinum*

## Abstract

Objectives: To explore the metabolic changes in zebrafish larvae after infection with *Mycobacterium marinum*, this study adopted a widely targeted metabolomic approach to analyze the changes in the overall metabolic profiles of zebrafish larvae infected for 5 days. Methods: Data were collected by liquid chromatography–tandem mass spectrometry (LC-MS/MS). Mass spectrometry data were processed using Analyst 1.6.3 and MultiQuant 3.0.3 software, and multivariate statistical analysis was carried out. The KEGG database, HMDB database, and CHEBI database were used to screen and identify differential metabolites, and metabolic pathway enrichment analysis was performed through KEGG pathways. Results: A total of 329 metabolites were detected, among which 61 differential metabolites were screened. Specifically, 41 metabolites, such as kynurenine, isoallolithocholic acid, 2′-deoxyguanosine, indole-3-carboxaldehyde, and L-lactic acid, were downregulated, while 20 metabolites, such as L-palmitoylcarnitine, myristoyl-L-carnitine, dodecanoylcarnitine, 2-isopropyl-malic acid, and 2-methylsuccinic acid, were upregulated. KEGG metabolic pathway enrichment analysis indicated that these differential metabolites were mainly involved in metabolic pathways such as pyrimidine metabolism, nucleotide metabolism, the pentose phosphate pathway, and purine metabolism. Conclusions: This study demonstrated that significant changes occurred in multiple metabolites and metabolic pathways in zebrafish larvae after infection with *M. marinum*. The research results have improved the understanding of zebrafish as a model organism in the field of *Mycobacterium* research and laid a solid foundation for subsequent metabolomic-related research using zebrafish.

## 1. Introduction

Tuberculosis (TB), caused by *Mycobacterium tuberculosis* (Mtb) [1], represents a global public health crisis. In 2023, there were 10.8 million new confirmed TB cases worldwide, with approximately 1.25 million deaths attributed to the disease [2], posing a severe threat to human health. Exploring suitable animal models to deeply investigate its pathogenesis and therapeutic strategies has always been a key task in scientific research. Zebrafish (*Danio rerio*), with characteristics of rapid reproduction, transparent embryos, and high genetic homology with humans, has become a classic model organism for mycobacterial infection research [3,4,5]. *M. marinum*-infected zebrafish exhibit granuloma structures and host immune responses highly similar to human tuberculosis [6,7], providing a unique platform for exploring host–pathogen interactions.

In recent years, metabolomics, an emerging field in systems biology, has provided a novel perspective for dissecting the mechanisms of host metabolic reprogramming during infection, leveraging its advantage of comprehensively and dynamically analyzing metabolite changes in organisms. Numerous studies have revealed the metabolic profiles in patients with pulmonary tuberculosis. Lau S.K. [8] identified four characteristic small-molecule metabolites in the plasma of pulmonary tuberculosis patients using metabolomics: 12R-hydroxy-5Z, 8Z, 10E, 14Z-eicosatetraenoic acid (12(R)-HETE), ceramide (d18:1/16:0), cholesterol sulfate, and 4α-formyl-4β-methyl-5α-cholesta-8-en-3β-ol. Their concentrations in plasma were significantly higher in pulmonary tuberculosis patients than in those with community-acquired pneumonia and healthy controls. Weiner [9] used gas chromatography–mass spectrometry (GC-MS) to identify approximately 400 small-molecule metabolites in the sera of tuberculosis patients, and found that the concentrations of 20 metabolites differed significantly between tuberculosis patients and healthy individuals, suggesting remarkable alterations in amino acid metabolism, lipid metabolism, bile acid metabolism, glutathione metabolism, and the urea cycle in tuberculosis patients.

A multicenter study [10] demonstrated that plasma levels of tryptophan and retinol were significantly reduced and kynurenine significantly elevated in tuberculosis patients. A metabolic signature model constructed based on the kynurenine/tryptophan ratio and retinol achieved an AUC of 0.97 for tuberculosis diagnosis, and the signature scores significantly decreased after anti-tuberculosis treatment, which was further validated in a non-human primate model of *Mtb* infection. Integrative analysis [11] of urinary proteomics and metabolomics screened out protein and metabolite biomarker panels that can efficiently distinguish pulmonary tuberculosis from other diseases, providing a non-invasive diagnostic approach. A hybrid metabolomic [12] strategy based on ultrahigh-performance liquid chromatography–tandem mass spectrometry identified 99 differential metabolites in the sera of tuberculosis patients, including a panel of highly efficient diagnostic biomarkers such as L-asparagine, L-glutamic acid, and arachidonic acid.

In contrast, studies in zebrafish models are limited. Existing research [13] shows that *M. marinum* infection significantly reshapes the amino acid metabolome of zebrafish larvae. Mass spectrometry-based quantification of 44 amine-containing small metabolites revealed that the majority of 30 metabolites were significantly reduced in infected zebrafish larvae compared to the control group, while five metabolites in the infected group—glutathione, putrescine, β-alanine, 5-hydroxylysine, and O-phosphoethanolamine—were increased.

Notably, the dual roles of metabolic reprogramming in mycobacterial infection, such as providing carbon sources for pathogens or activating host antibacterial pathways, have been widely confirmed in mammalian models, but studies on homologous mechanisms in zebrafish remain entirely unexplored. For example, glutamine metabolism inhibition exhibits dual immunomodulatory and antibacterial activities against *Mtb*: in a murine TB model [14], treatment with JHU083 leads to reduced levels of immunosuppressive myeloid cells, enhanced effector T-cell levels, and increased production of citrulline and NO. Importantly, although JHU083 possesses both direct antibacterial effects and immunomodulatory activity, it primarily functions as a host-directed immunotherapy in murine TB models. Another study [15] has revealed how the TB pathogen *Mtb* exploits the host’s tryptophan metabolic pathway to delay T-cell immune responses: specifically, high levels of the tryptophan metabolite kynurenine were found to inhibit T-cell infiltration in the lungs of TB patients, whereas targeting the aryl hydrocarbon receptor (AhR) and indoleamine 2,3-dioxygenase 1 (IDO1) accelerated T-cell immune responses, thereby enhancing host control of *Mtb* infection. Additionally, the unique metabolic adaptations of zebrafish may lead to biases in cross-species mechanistic extrapolation, urgently requiring targeted metabolomic data support.

Despite the significant value of these research achievements in demonstrating the role of metabolomics in revealing host defense mechanisms and advancing the development of novel anti-tuberculosis therapies, the systematic analysis of metabolic characteristics at different stages of *M. marinum* infection in zebrafish remains notably insufficient. Using zebrafish as a model organism, this study employed broadly targeted metabolomic technology to comprehensively analyze the dynamic changes in the metabolic profile of zebrafish following *M. marinum* infection, aiming to provide deeper and more systematic metabolic insights for understanding the pathogenesis of tuberculosis and developing new treatment strategies. While long-term dynamic research remains a future direction, this study for the first time establishes a metabolic fingerprint of the zebrafish–*M. marinum* infection model at 5 days post-infection (5 dpi), laying an important foundation for subsequent longitudinal studies across multiple time points.

## 2. Materials and Methods

### 2.1. Zebrafish Cultivation

AB wild-type zebrafish are reared at the Zebrafish Experimental Platform in the Research Building of the Shanghai Public Health Clinical Centre. The zebrafish used in this experiment were reared in a standard recirculating water system (32 °C, pH 5–6). The dark and light conditions were set for 10:14 h. Feedings were given twice per day with an appropriate amount of *Artemia nauplii*. The zebrafish animal model in this study was also approved by the Animal Ethics Committee of the Shanghai Public Health Clinical Centre (approval 2019-A016-01).

### 2.2. Zebrafish Embryo Infection and Sample Collection

Zebrafish embryos were infected with *M. marinum* (ATCC BAA-535) at 3 days post-fertilization (3 dpf) via microinjection into the duct of Cuvier, with an infection dose of 250 CFU. The infection protocol was adapted from previously described methods [16]. At 5 days post-infection (dpi), larvae were euthanized by immersion in 1% tricaine (MS-222). Larvae were collected into microcentrifuge tubes, with each sample consisting of 50 larvae [17] and four parallel samples per experimental group. All samples were collected and stored at −80 °C until use.

### 2.3. Widely Targeted Metabolomics and Data Analysis

#### 2.3.1. Pre-Processing

To each sample, 500 μL of ice-cold (−20 °C) 70% methanol–water extraction buffer was added, followed by vortexing for 3 min to ensure thorough mixing. Samples were then centrifuged at 12,000× *g* for 10 min at 4 °C. A 300-μL aliquot of the resulting supernatant was transferred to a fresh 1.5 mL microcentrifuge tube. Following incubation at −20 °C for 30 min to facilitate protein precipitation, samples were centrifuged again under identical conditions (12,000× *g*, 10 min, 4 °C). Finally, 200 μL of the clarified supernatant was carefully transferred through a protein precipitation plate and stored at −20 °C until LC-MS/MS analysis.

#### 2.3.2. LC-MS/MS Analytical Conditions

The sample extracts were analyzed using an LC-MS/MS system (UPLC, ExionLC ADˈ; MS, QTRAP System) (Sciex, Framingham, MA, USA) by Wuhan Metware Biotechnology Co., Ltd. Chromatographic separation was achieved using either a Waters ACQUITY UPLC HSS T3 C18 column with a gradient of 0.05% formic acid in water and acetonitrile or an ACQUITY UPLC BEH amide column with a gradient of 10 mM ammonium acetate and 0.3% ammonia in water and 90% acetonitrile/water. Mass spectrometry employed electrospray ionization (ESI) at 550 °C with ±5.5 kV ionization voltage and 35 psi curtain gas.

#### 2.3.3. Data Analysis  

Mass spectrometry data were processed using MultiQuant 3.0.3 software. Unsupervised principal component analysis (PCA) was performed using the prcomp function in R (v. 3.5.1), with data subjected to unit variance scaling prior to analysis to ensure equal contribution of variables. Hierarchical cluster analysis, Pearson correlation coefficients, HCA (hierarchical cluster analysis) results of samples and metabolites are presented as heatmaps with dendrograms, while Pearson correlation coefficients (PCCs) between samples were calculated by the cor function in R and are presented as only heatmaps. Both HCA and PCC were carried out using the R package ComplexHeatmap (v. 2.7.1.1009). For HCA, normalized signal intensities of metabolites (unit variance scaling) are visualized as a color spectrum. Significantly regulated metabolites between groups were identified by VIP  ≥  1 and absolute log_2_FC (fold change)  ≥  1. VIP values were extracted from OPLS-DA results, which also contained score plots and permutation plots, which were generated using the R package MetaboAnalystR. Metabolites were annotated using the Kyoto Encyclopedia of Genes and Genomes (KEGG) database, and KEGG pathway enrichment analysis was performed based on the differentially expressed metabolites.

## 3. Results

### 3.1. Metabolic Profile Changes in Zebrafish Larvae upon Infection

**Figure 1:**
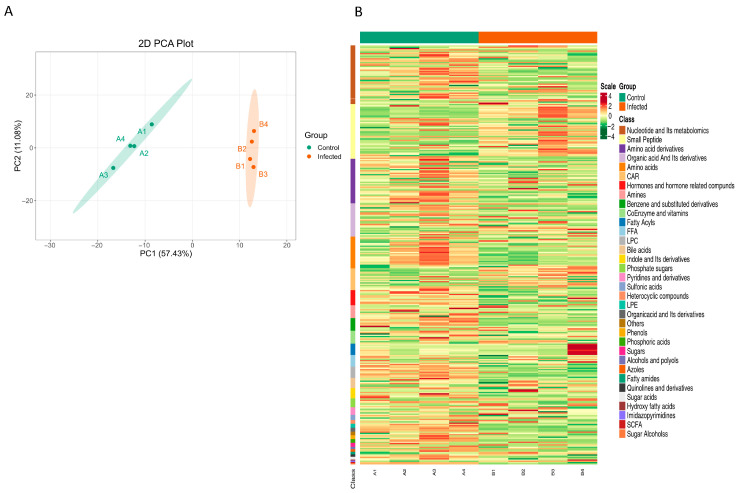
Global metabolic profiles of zebrafish larvae after *M. marinum* infection. (**A**) Analysis of uninfected zebrafish larvae (control) or larvae infected with *M marinum* at 3 days post-fertilization (3 dpf). All larvae were collected at 5 days post-infection (5 dpi) and then measured by LC-MS/MS. (**B**) Principal component analysis (PCA) plot of zebrafish larvae in the infected and control groups. Each point represents a sample, n = 4. dpf: days post-fertilization.

In the zebrafish larvae infection model, samples were analyzed using widely targeted metabolomic technology (LC-MS/MS), and a total of 329 metabolites were detected. Principal component analysis (PCA, Figure 1A) first revealed significant separation between the infected group and the control group along PC1 (57.43% variance contribution) and PC2 (11.08% variance contribution), visually demonstrating the remodeling effect of infection on the overall metabolic profile. Further hierarchical clustering analysis (Figure 1B, Table S1) detailed the differences in abundance of functional categories of differential metabolites. The results demonstrated significant upregulation of metabolites in categories such as nucleotides and their metabolomics, small peptides, and benzene and substituted derivatives, suggesting enhanced energy metabolism and signal molecule synthesis during infection. Conversely, metabolites including short-chain fatty acids (SCFAs), sugar alcohols, and free fatty acids (FFAs) were markedly downregulated, potentially reflecting increased host energy expenditure or disrupted gut microbiota metabolism. Additionally, bile acids and hormones and hormone-related compounds exhibited moderate downregulation, implying impaired endocrine regulation. This multilayered metabolic imbalance provides critical molecular insights into the pathological mechanisms underlying infection.

### 3.2. Screening of Differential Metabolites

**Figure 2:**
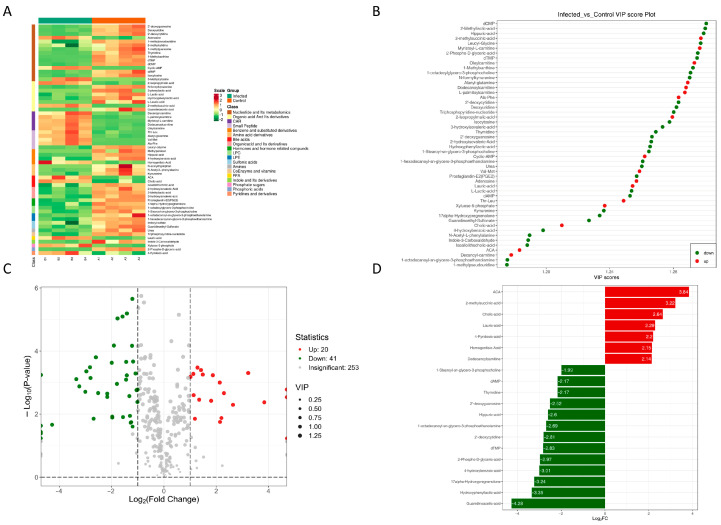
Analysis of differential metabolites in zebrafish larvae infected with *M. marinum*. (**A**) Heatmap of differentially expressed metabolites, where the original contents of the metabolites are normalized row-wise using unit variance scaling (UV scaling). (**B**) VIP score plot of differentially expressed metabolites, showing the 50 metabolites with the highest VIP values in the OPLS-DA model. (**C**) Volcano plot of differentially expressed metabolites, applying a triple-screening criterion of VIP, fold change (FC), and *p*-value. The ordinate represents the significance level of differences (−log_10_ *p*-value), and the size of the dots indicates the VIP value. (**D**) Bar chart of the top 20 metabolites ranked by fold change, with the abscissa representing VIP values and the ordinate listing metabolites. Red bars denote upregulated metabolites, while green bars indicate downregulated metabolites. FC = fold change (infected/control), log_2_-transformed.

Based on the above metabolic profile differences, we adopted multidimensional criteria to screen differential metabolites, prioritizing metabolites with VIP≥1 (Figure 2B–D) that significantly contributed to intergroup separation combined with |log_2_FC| ≥ 1 (FC ≥ 2 or ≤0.5) and *p* < 0.05 (Figure 2C). A total of 61 differential metabolites were finally identified (20 upregulated, 41 downregulated). In the volcano plot of Figure 2C (Table S2), red points (upregulated, 20) and green points (downregulated, 41) are significantly distributed in the region with |log_2_FC| ≥ 1 and −log_10_ (*p*-value) ≥ 2, while gray points (253) represent metabolites with no significant difference, visually presenting infection-induced changes in metabolite abundance. In the VIP score plot of Figure 2D, the 10 metabolites with the highest VIP values contributed most to intergroup separation. These metabolites were involved in immune-related pathways, including tryptophan metabolism intermediates, pro-inflammatory lipids, and energy metabolism pathways such as glycolytic substrates and glutamine, reflecting the imbalance between energy supply and immune response under infection. The hierarchical clustering heatmap (Figure 2A, Table S3) further shows the abundance patterns of differential metabolites classified by functional categories such as nucleotides and their metabolites, organic acids and their derivatives, and amino acid derivatives. Compared with the control group, metabolites including kynurenine, isoallolithocholic acid, 2′-deoxyguanosine, 2′-deoxyguanosine, indole-3-carboxaldehyde, and L-lactic acid were downregulated in the infected group, while L-palmitoylcarnitine, myristoyl-l-carnitine, dodecanoylcarnitine, 2-isopropyl-malic acid, and 2-methylsuccinic acid were upregulated.

**Figure 3:**
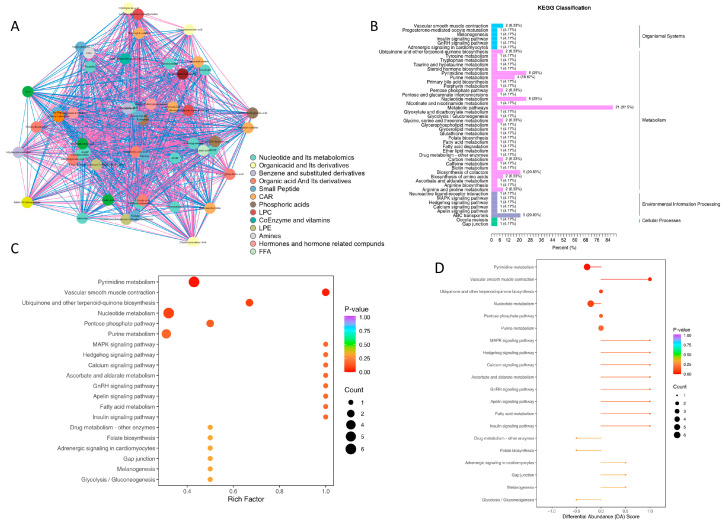
KEGG metabolic pathway enrichment analysis of differential metabolites. (**A**) Correlation network of differential metabolites, where nodes represent significantly differential metabolites. Node size is positively correlated with connectivity (degree): red lines indicate positive correlations, blue lines indicate negative correlations, and line thickness reflects the absolute value of correlation coefficients. (**B**) KEGG classification map of differential metabolites. The numbers denote the quantity of differential metabolites annotated to each pathway, and the values in parentheses represent the proportion of differential metabolites in this pathway to the total number of KEGG-annotated differential metabolites. (**C**) KEGG enrichment plot of differential metabolites. The *x*-axis shows the Rich factor for each pathway, and the *y*-axis lists pathway names. Node color corresponds to the  *p*-value, while node size represents the number of differential metabolites enriched in the pathway. (**D**) Differential abundance score plot for all differential metabolic pathways. The size of dots at the endpoints of line segments reflects the number of differential metabolites in each pathway, and color intensity corresponds to the *p*-value.

There were synergistic or mutually exclusive relationships between different metabolites. Correlation analysis helped measure the metabolic proximities between significantly differential metabolites, facilitating a deeper understanding of the regulatory relationships between metabolites during biological state changes. Pearson correlation analysis was performed on the differentially significant metabolites screened according to the criteria. Functional network analysis (Figure 3A) showed that there were strong connections between nucleotide metabolites and organic acid derivatives, indicating close metabolic interactions. Among them, metabolites of the same functional category such as nucleotides and small peptides had dense internal connections, indicating that similar metabolites were coordinately regulated under infection, such as the overall activation or inhibition of nucleotide metabolism pathways. Between different functional categories, nucleotides and organic acid derivatives showed negative correlations, suggesting that in the infection-induced metabolic reprogramming, energy metabolism-related nucleotides, and immune metabolism-related organic acid derivatives such as kynurenine precursors might have antagonistic regulation, reflecting the host’s metabolic–immune balance adjustment to infection. In addition, nucleotide metabolites located at central nodes with high connectivity might be key hubs for infection metabolic regulation, and their abundance changes could affect multiple pathways such as glycolysis and immune signaling through the network.

### 3.3. KEGG Functional Annotation and Metabolic Pathway Analysis of Differential Metabolites

Subsequently, we performed KEGG annotation on the differential metabolites (Figure 3B) and found that they were predominantly enriched in metabolic pathways, accounting for 89.8%, covering core modules such as amino acids, lipids, and energy metabolism. Pathway enrichment analysis (Figure 3C,D) revealed that significantly enriched pathways with *p* < 0.05 included pyrimidine metabolism, vascular smooth muscle contraction, and ubiquinone and terpenoid quinone biosynthesis. Among them, the pyrimidine metabolism pathway had the highest DA score of 1.8, making it a core pathway in infection-related metabolic reprogramming. It is speculated to be involved in energy supply and immune escape regulation during mycobacterial infection.

## 4. Discussion

This study employed a widely targeted metabolomic approach to reveal significant changes in metabolites and metabolic pathways in zebrafish larvae after infection with *M. marinum*. These findings have multiple potential connections with TB research, providing new insights into the pathogenesis of *Mtb*-associated diseases.

At the metabolite level, 61 differential metabolites were identified, including kynurenine and L-lactic acid, whose patterns of change are similarly reported in TB studies. For example, kynurenine, a key product of tryptophan metabolism, plays a critical role in TB infection [18,19]. Tryptophan has been reported as a biomarker for TB patients in multiple studies [20,21,22] involving diagnosis and treatment. Upon infection of macrophages by *Mtb*, indoleamine 2,3-dioxygenase (IDO) is activated, promoting the conversion of tryptophan to kynurenine. Kynurenine not only inhibits T-cell function to help pathogens escape immune surveillance but also shows a negative correlation with T-cell effector cytokines IFN-γ and TNF-α, reflecting the chronic inflammatory state of the body [15]. The downregulation of kynurenine in this study may indicate a unique pattern of immunometabolic regulation in zebrafish larvae after *M. marinum* infection, differing from the immunosuppression-dominated state in TB infection, which might be related to the unique immune defense mechanisms of zebrafish. Interestingly, while kynurenine is typically upregulated in human TB due to IFN-γ-induced IDO1 activity, we observed a decrease in zebrafish. This discrepancy may reflect the complexity of immune regulation and metabolic networks, for which we postulate several possibilities. First, species-specific differences in metabolic pathway regulation exist. In mammals, elevated kynurenine serves as a hallmark of immune tolerance in chronic infections, mediated by IDO1 activity in myeloid cells. Previous studies have shown that although zebrafish possess a functional tryptophan-kynurenine metabolic machinery, their metabolite kinetics and tissue-specific roles differ significantly. For instance, kynurenine metabolism exhibits more compartmentalized characteristics, with local elevation observed under neuroinflammatory conditions [23]. Furthermore, downstream metabolite kynurenic acid (KYNA) plays unique roles in fish development and stress adaptation [24]. Second, metabolic plasticity in different disease contexts further exacerbates this complexity. Plasma tryptophan and kynurenine levels are reduced in patients with major depressive disorder (MDD) and bipolar disorder (BD) [25], whereas individuals with obsessive–compulsive disorder (OCD) show elevated kynurenine metabolites [26], indicating that the same metabolite can exhibit contradictory changes across diseases. Additionally, experimental model characteristics may influence the results. Our study used only zebrafish larvae at a single time point, while human studies primarily rely on blood samples from adults. The difference between whole-larva tissues in zebrafish and human tissue samples may lead to variations in metabolite distribution and regulation.

L-lactic acid showed a downward trend in this study. In TB infection, glycolysis in host cells is enhanced and lactic acid production increases due to local hypoxic environments [27,28]. Lactic acid significantly reduces the concentrations of TNF and IL-1β produced by human macrophages against *Mtb* while improving the bacterial clearance rate in *Mtb*-infected human macrophages, at least partially by promoting autophagy. The glycolytic product lactic acid exerts a negative feedback effect on macrophages, leading to weakened glycolytic shifts and reduced pro-inflammatory cytokine production after subsequent stimulation [29]. In the zebrafish model, L-lactic acid plays a multifaceted role in host responses to *Mtb*. Monocarboxylate transporter 4 (Mct4), responsible for lactate export, is upregulated in *Mtb*-infected macrophages and in TB patients. Mct4 facilitates *Mtb* survival by preventing intracellular lactate accumulation and activating the NF-κB p65–IL-10 axis, a mechanism confirmed both in *vitro* and in *vivo* [30]. Lactate also recruits and polarizes macrophages toward an M2-like phenotype, promoting angiogenesis through VEGF, TGF-β, and IL-10 signaling, as demonstrated in zebrafish xenografts [31]. Moreover, lactate modulates inflammation via histone H3K18 lactylation, which—together with elevated reactive oxygen species (ROS)—enhances neutrophil recruitment and inflammatory responses [32]. Notably, excessive TNF-induced mitochondrial ROS (mROS), a key driver of pathological macrophage necrosis in TB, may also be influenced by lactate metabolism [33]. Finally, real-time imaging using the Laconic biosensor in zebrafish reveals rapid lactate surges following tissue injury, indicating metabolic reprogramming that may parallel immune activation during *Mtb* infection [34]. Collectively, these findings suggest that lactate not only reflects but also actively shapes the metabolic and immune landscape of TB pathogenesis.

Additionally, other differential metabolites also show potential associations with metabolic mechanisms of infectious diseases. Carnitine (CAR), a key carrier of fatty acid β-oxidation, plays an important regulatory role in host fatty acid metabolism [35,36]. The upregulation of differential carnitine metabolites (L-palmitoylcarnitine, myristoyl-L-carnitine) may reflect the reprogramming of lipid metabolism in zebrafish larvae after infection. In TB infection, mycobacteria rely on host fatty acid oxidation for energy, hijacking the carnitine transport system to obtain long-chain fatty acids (such as palmitic acid) to support their mitochondrial metabolism [37]. The significant upregulation of L-palmitoylcarnitine in this study may suggest that *M. marinum* utilizes host lipid resources through similar mechanisms while the host attempts to maintain energy supply via enhanced fatty acid oxidation to cope with infection stress. Indole-3-carboxaldehyde, an important byproduct of tryptophan metabolism, may be downregulated due to insufficient IDO pathway activation or gut microbiota dysregulation, and it can promote tumor immunotherapy [38]. As an intermediate of leucine metabolism, the upregulation of 2-isopropyl-malic acid may be related to the activation of branched-chain amino acid (BCAA) metabolism [39,40,41]. Leucine can regulate macrophage polarization through the mTOR pathway [42], and its deficiency inhibits T-cell proliferation [43]. The accumulation of this metabolite in zebrafish may indicate enhanced BCAA catabolism induced by infection to provide energy substrates or participate in inflammation regulation.

The downregulation of bile acid metabolites may be related to liver detoxification dysfunction or gut microbiota dysregulation [44]. Lithocholic acid derivatives have antibacterial activity [45], and a reduction in isoallolithocholic acid in zebrafish may weaken the host’s direct killing ability against pathogens or reflect increased metabolic load in the hepatobiliary system after infection, leading to imbalance in bile acid synthesis and secretion. Notably, bacterial infection-induced gut microbiota dysbiosis may directly influence metabolic profiles, as evidenced by studies in TB patients. For example, Maji found that active pulmonary TB patients showed enrichment of butyrate/propionate-producing bacteria, e.g., Faecalibacterium, Roseburia, and reduced biosynthesis of vitamins and amino acids, indicating that the microbiota mediates shifts in short-chain fatty acid (SCFA) metabolism [46]. Similarly, Zhang [47] reported decreased α-diversity of the gut microbiota in TB patients accompanied by altered levels of SCFAs and amino acids, which are key metabolites linking microbial activity to host energy homeostasis. Wang [48] further confirmed that PTB patients had reduced SCFA production, with fecal metabolomic changes correlating with the abundance of Bacteroides and Firmicutes. In addition, studies have found that the abundance of *Akkermansia muciniphila* in the intestines of patients with active tuberculosis is significantly reduced [49]. This bacterium can produce palmitoleic acid, which inhibits the expression of the TNF gene through epigenetic modification. A cohort study of 6512 individuals showed that variations in the IFNAR1 gene can inhibit the colonization of this bacterium, leading to a decrease in palmitoleic acid production and an increased susceptibility to tuberculosis. In the context of our study, the observed alterations in L-lactic acid and bile acids may reflect similar microbiota-metabolism cross talk.

From the perspective of metabolic pathways, pathways closely related to the survival and proliferation of mycobacteria, such as pyrimidine metabolism and nucleotide metabolism, were significantly enriched after zebrafish infection. Pyrimidine metabolism plays an important role in TB infection [50] and serves as a metabolic checkpoint during mycobacterial adaptation to non-growing states [51]. As the basis of nucleic acid synthesis, its changes may reflect the competition for nucleotide resources between the host and pathogens, while differences in nucleotide metabolism may be related to zebrafish’s response to pathogen invasion and interference with their drug resistance mechanisms. Functional network analysis showed strong connections between nucleotide metabolites and organic acid derivatives, suggesting that this metabolic interaction may be exploited by pathogens to manipulate host metabolic balance, such as acquiring nucleotide synthesis precursors through organic acids. In the KEGG differential abundance scoring, the pyrimidine metabolism pathway had the highest DA score, making it a core pathway in infection-related metabolic reprogramming, presumably involved in energy supply and immune escape regulation during mycobacterial infection. These results elucidate the mechanism by which infection affects host immune responses through metabolic pathways, providing targets for subsequent infection–metabolism interaction studies and forming a complete logical chain from metabolic profile differences to functional pathway analysis.

In summary, the diverse changes in differential metabolites in this study reveal the synergistic remodeling of energy metabolism (lipids/nucleotides), immunometabolism (tryptophan/BCAA), and detoxification functions (bile acids) in zebrafish after *M. marinum* infection. This study focused on the acute phase within 5 dpi of zebrafish larvae infected with *M*. *marinum*. However, considering that tuberculosis is a chronic infectious disease, it is also crucial to investigate how host metabolism continues to evolve in the late infection stage. Metabolic adaptations during chronic infection may involve sustained shifts toward lipid utilization, glycolytic inhibition, and altered redox balance, which are associated with immune cell persistence and granuloma maintenance. The granulomatous microenvironment drives host metabolic reprogramming toward lipid utilization through nutrient limitation and hypoxic conditions. The study has shown that bacterial cell-wall lipids (e.g., trehalose-6,6′-dimycolate, TDM) actively regulate host angiogenesis, a process that requires substantial lipid support [52]. The mTORC1 signaling pathway protects macrophages from death by maintaining infection-induced mitochondrial energy metabolism, a protective effect dependent on energy generated via glycolysis [53]. However, during the chronic phase, the hypoxic milieu within granulomas drives bacteria into a dormant state, whereby host cells may downregulate glycolysis to match the bacteria’s low metabolic demands [54]. These long-term metabolic changes may facilitate bacterial survival strategies, such as dormancy or evasion of host immunity. Future studies using extended time points or adult zebrafish will be critical for mapping host metabolic dynamics throughout infection.

## Data Availability

Data supporting this study are available from the corresponding author upon reasonable request.

## Supplementary Materials

The following supporting information can be downloaded at https://www.mdpi.com/article/10.3390/metabo15070449/s1. Table S1. Raw data table of all metabolites. Table S2. Data of screening differential metabolites, including VIP, *p*-value, FDR, FC, and log2FC parameters; Table S3. Data of 61 differential metabolites.
