# Supplementary material for: Metabolic Changes in Zebrafish Larvae Infected with Mycobacterium marinum: A Widely Targeted Metabolomic Analysis
Source: Metabolites. 2025 Jul 4;15(7):449. doi: 10.3390/metabo15070449 (PMC12298622; doi:10.3390/metabo15070449)
Supplement: Supplementary file 1 [file metabolites-15-00449-s001.zip › Supplementary Materials.pdf]

Supplementary Materials

Supplementary Table 1. Raw data table of all metabolites

Supplementary Table 2. Data table for screening differential metabolites, including VIP, P - value, FDR, FC, Log2FC parameters

Supplementary Table 3. Data table of 61 differential metabolites
